# The relationship between mindfulness and suboptimal health status: a chain/serial mediation model

**DOI:** 10.3389/fpsyg.2024.1354975

**Published:** 2024-03-20

**Authors:** Jingyue Liang, Xiaoshuo Zhang, Yuzheng Wang

**Affiliations:** ^1^CAS Key Laboratory of Mental Health, Institute of Psychology, Beijing, China; ^2^Department of Psychology, University of Chinese Academy of Sciences, Beijing, China; ^3^Department of Undergraduate College, Beijing Anzhen Hospital, Capital Medical University, Beijing, China

**Keywords:** mindfulness, suboptimal health, social support, stress, chain/serial mediation

## Abstract

**Background:**

Suboptimal health status (SHS) represents a third state between health and disease and often progresses into chronic conditions, negatively impacting an individual’s well-being. Studies have shown that mindfulness has a beneficial effect on various SHS symptoms. This study aims to explore the influence of mindfulness on SHS and its underlying mechanisms, with a particular focus on examining the mediating roles of stress and social support.

**Methods:**

A total of 173 healthy Chinese college or graduate students, with an average age of 21.85 years, participated in this study. Measurements were taken using the Five Factor Mindfulness Questionnaire, the Sub-Health Measurement Scale, the Perceived Stress Scale, and a self-constructed scale that included demographic information. The PROCESS plugin for SPSS was used to assess mediating effects.

**Results:**

Significant correlations were found among SHS, social support, mindfulness, and perceived stress (|*r*| = 0.38–0.85, *p* < 0.01). Specifically, mindfulness showed a significant positive correlation with SHS and social support (*r* = 0.38–0.77), while perceived stress was significantly negatively correlated with mindfulness, social support, and SHS (|*r*| = 0.45–0.85). Perceived social support was positively associated with SHS (*r* = 0.65). Furthermore, social support and perceived stress partially mediated the influence of mindfulness on SHS. Additionally, a sequential mediation effect of perceived social support and stress in the relationship between mindfulness and SHS was supported.

**Conclusion:**

The cultivation of trait mindfulness may be advantageous for individuals’ sub-health. Perceived social support and perceived stress are important underlying mechanisms contributing to this effect.

## Introduction

Suboptimal health status (SHS) is considered an intermediate state between health and disease, often referred to as the “third state.” According to the Clinical Guidelines of Chinese Medicine on Sub-health issued by China Association of Chinese Medicine (2006), SHS is defined as “a state in which the human body is in between health and disease. Individuals in a suboptimal health state cannot meet the standards of health and exhibit symptoms of reduced vitality and diminished function and adaptability for a certain period. However, they do not meet the clinical or subclinical diagnostic criteria for diseases according to modern medicine.” While the symptoms of SHS are considered in the medical domain, it is closely intertwined with various factors such as the social environment, economic and cultural factors, psychological factors, and individual constitution when viewed comprehensively.

Research has indicated that a state of sub-health is mainly caused by a combination of factors such as high mental and psychological stress, irregular diet, and disrupted sleep patterns (Huali, 2021). Although SHS may not pose a severe threat to an individual’s health in the short term, it has significant impacts on people’s daily lives and work. Therefore, addressing and managing SHS is of great significance. Meta-analytic studies have confirmed that mindfulness-based interventions (MBI) rooted in mindfulness theory, such as Mindfulness-Based Stress Reduction (MBSR) and Mindfulness-Based Cognitive Therapy (MBCT), can effectively alleviate stress and SHS-related symptoms such as anxiety, depression, worry, panic, and chronic illnesses. These interventions have also been shown to significantly improve quality of life and overall well-being (Querstret et al., 2020; Zhang et al., 2021).

SHS presents a complex array of symptoms influenced to some extent by individual factors such as age, adaptability, immune function, and socio-cultural context. Common symptoms include reduced vitality, diminished reactivity, decreased adaptability, and lowered immunity, with associated problems in areas like physical well-being, appetite, sleep, and interpersonal relationships (Zhao and Song, 2002). Mindfulness involves focusing on present objectives and cultivating awareness of each moment’s experiences without judgment. Mindfulness practices have been associated with positive health effects (Ludwig, 2008). Previous research has shown that MBIs, such as meditation, yoga, and stress reduction, have significant positive effects on many SHS symptoms. For instance, MBI can reduce pain perception, enhance flow, improve function, and increase happiness (Majeed et al., 2018). MBSR has been found to enhance immune function and improve sleep quality to some extent (Wang et al., 2014). Mindfulness training can also influence metabolic states (Chen et al., 2020) and has both short-term positive effects on health-related behaviors and the overall negative effects on impulsive eating and binge eating (Ruffault et al., 2017). Furthermore, higher levels of dispositional mindfulness are associated with lower levels of fatigue. Similar graded relationships between dispositional mindfulness and depression symptoms, poor sleep quality, childhood adversity, and chronic diseases have been observed (Whitaker et al., 2019). Based on the available research evidence, Hypothesis 1 is proposed:

Higher levels of trait mindfulness are positively associated with suboptimal health levels.

While previous research has supported the positive impact of mindfulness on SHS symptoms, further investigation is needed to understand the mechanisms involved. Mindfulness can increase the perception of social support, and social support mediates the positive effects of mindfulness. Perceived social support is widely defined as the perception that an individual is cared for by others and has a reliable social network to turn to when needed. It encompasses various sources of support, including family, friends, and significant others (Taylor, 2011). Social support plays a protective role in an individual’s physical and mental health. For example, Amiya et al. (2014) found that family support has a protective effect on users of pharmaceuticals against depression and other mental health issues. Gu et al. (2016) discovered that creating a favorable social environment and increasing the internal perception of support and workplace support are crucial for improving the suboptimal health symptoms of bedside nurses. There is evidence that mindfulness can positively predict perceived social support, possibly because mindfulness allows individuals to focus on their current experiences and be aware of the support they receive from their social networks (Kuhl and Boyraz, 2016). Sun et al. (2019) found that perceived social support partially mediates the relationship between mindfulness and burnout among special education teachers. Additionally, research has shown that perceived peer relationships mediate the effects of mindfulness on stress and anxiety (Wen et al., 2021). Therefore, we hypothesize that social support is one of the mechanisms through which mindfulness affects suboptimal health, leading to Hypothesis 2:

Perceived social support mediates the relationship between mindfulness and suboptimal health.

Stress is a perception that a situation or event exceeds one’s coping resources (Folkman et al., 1987; Lazarus and Folkman, 2020). Stress is associated with various issues affecting human quality of life, such as autoimmune diseases, migraines, obesity, muscle tension and back pain, high cholesterol, coronary heart disease, hypertension, and stroke (Sharma and Rush, 2014). Stress is usually a specific reactive process that can be explicitly measured by psychological and physiological indicators. In contrast, sub-health status is a more generalized concept that encompasses a range of non-specific symptoms and experiences. Stress may have a combined effect on an individual’s SHS through the endocrine system, psychological states, behavioral patterns, and social and environmental factors. Chronic or excessive stress can cause chronic hormonal imbalances, such as elevated cortisol levels, which can lead to decreased immune function, sleep disorders, and energy metabolism problems, all of which are common symptoms of SHS. Stress also affects an individual’s mood and cognitive functioning, leading to reduced concentration and memory, emotional instability, and so on. In addition, individuals under prolonged high-pressure situations may develop unhealthy lifestyle habits, such as irregular diet and lack of exercise, further exacerbating the subhealth state. Therefore, effective management of stress is crucial to preventing and improving SHS. MBIs focus on aspects like awareness of the present moment, mind–body connection, attention control, non-judgmental thinking, and bodily sensations (Bamber and Schneider, 2016). These interventions include various mindfulness meditation techniques, such as mindful breathing, body scanning, mindfulness of emotions and thoughts, and awareness of sensory experiences. One of the most popular mindfulness interventions is MBSR. The efficacy of mindfulness-based techniques in alleviating stress has received extensive research support (Sharma and Rush, 2014; Bamber and Schneider, 2016). Mindfulness can buffer the negative impact of stress by reducing negative evaluations of stress and, ultimately, mitigating the adverse effects of stress on individuals, including SHS symptoms. Based on these findings, we propose Hypothesis 3:

Perceived stress mediates the relationship between mindfulness and suboptimal health.

Cohen and Wills (1985) have extensively defined social support as a “buffering effect.” It can prevent the harmful effects of stress by manipulating the stress source itself, changing the meaning individuals assign to stress, or improving emotional responses triggered by stress (Waqas et al., 2018). Therefore, it can be inferred that social support can directly improve an individual’s suboptimal health status and indirectly benefit their physical and mental health by alleviating stress. However, some research has found that while psychological resilience mediates the relationship between mindfulness and anxiety, as well as between mindfulness and depression, social support does not mediate the relationship between mindfulness and psychological resilience (Gu et al., 2022). The evidence for the indirect impact of social support on other psychological variables is still limited. Therefore, we propose Hypothesis 4:

Mindfulness has a positive effect on suboptimal health through a chain-mediation pathway involving social support and perceived stress.

This study highlights the pivotal role of mindfulness in enhancing SHS, mediated by perceived social support and stress reduction. It underscores the utility of mindfulness practices in health promotion and disease prevention, filling a vital knowledge gap and guiding future targeted interventions. This research marks a significant contribution to understanding and optimizing mindfulness’s impact on health and well-being.

## Materials and methods

### Participants

We conducted a sample size calculation using Gpower 3.1.9.7, with a significance level set at α = 0.05, an effect size of 0.25, and a statistical power (1 – β) of 0.95. The required sample size was determined to be 73. This study was conducted using the Wenjuanxing web platform,^1^ utilizing convenience sampling methods. Participants were selected based on the following criteria: currently enrolled in college or graduate programs, in good health, free of any chronic disease or psychological disorder, and able to fluently understand and complete the questionnaire. Exclusion criteria were participants who did not meet the inclusion criteria, responding without seriousness and providing incomplete or incorrect personal information. Prior to participation, individuals were provided with information about the study’s purpose, its voluntary nature, confidentiality measures, and the approximate time required to complete the survey. Informed consent was obtained online, with participants acknowledging their understanding and agreement to participate before proceeding to the questionnaire.

The collected data were analyzed using statistical software. A sample size calculation was conducted using Gpower 3.1.9.7, setting a significance level at α = 0.05, an effect size of 0.25, and a power of 0.95, which determined a required sample size of 73. Ultimately, we collected 173 valid questionnaires (69.20%). Participants had an average age of 21.85 years (SD = 3.34), with 107 males (61.80%) and 66 females (38.2%), as shown in Table 1.

**Table 1:** Socio-demographics characteristics of the participants in the survey (*n* = 173).

Variable		*n*	%
Sex	Male	107	61.8
	Female	66	38.2
Age	21.85 ± 3.34	–	–
Level of education	Junior high school and below	2	1.2
	High School or Junior College	14	8.1
	University or college	150	86.7
	Graduate students and above	7	4.0
Marital status	Unmarried	143	82.7
	Married	27	15.6
	Divorced	3	1.7
*Per capita* monthly household income	Less than 2500RMB	8	4.6
	2,500–5,000 Yuan	59	34.1
	5,001 Yuan-7500 Yuan	73	42.2
	More than 7,500 yuan	33	19.1

### Procedure

This study utilized a cross-sectional survey design to examine the predictive effect of mindfulness on suboptimal health, as well as the mediating roles of social support and perceived stress among Chinese university students and graduate students. The independent variable for the investigation was mindfulness, which refers to the participants’ awareness and attention to present moments in a non-judgmental manner. The dependent variable was suboptimal health status, characterized by a state between health and illness, where individuals experience symptoms of discomfort or declined functioning without a diagnosed illness. Social support and perceived stress were investigated as mediating variables, with social support defined as the perception of being cared for and having assistance available from other people, and perceived stress referring to the degree to which situations in one’s life are appraised as stressful. Data collection was carried out using the Wenjuanxing web platform (see text footnote1). The main content of the questionnaire included three scales and one question related to social support. The study procedures were approved by the Ethics Committee of the Institute of Psychology, Chinese Academy of Sciences. After obtaining informed consent, participants were directed to the online survey hosted on the Wenjuanxing platform. The survey was designed to be completed within 3–6 min, starting with the demographic question, followed by the mindfulness scale, social support questionnaire, and perceived stress scale, in that order. Descriptive analyses, correlation and reliability analyses of the data were performed using SPSS 26.0. Mediation effects were verified using the PROCESS plugin for SPSS.

### Measures

The questionnaire initially collected demographic information (gender, age, and ethnicity), lifestyle habits (sleep, diet, physical activity), and social support. The social support item was as follows: “When encountering difficulties, I can obtain help and support from family, friends, and other sources” (rated on a 5-point scale: 1 completely disagree to 5 completely agree).

### Five facet mindfulness questionnaire short form

The FFMQ-SF consists of 20 items and is a multidimensional scale used to measure mindfulness (Chung et al., 2016). It employs a Likert 5-point scoring system with scores ranging from 1 never or very rarely true to 5 often or always true. The scale measures five facets of mindfulness, namely Observing (Items 3, 7, 9, 15), Describing (Items 1, 13, 16, 18), Acting with Awareness (Items 2, 6, 8, 19), Non-judging (Items 5, 12, 14, 20), and Non-reactivity (Items 4, 10, 11, 17). The items for the facets of Acting with Awareness and Non-judging are reverse-scored. In this study, we collected and used the total score of the FFMQ-SF. In this study, the Cronbach’s α for the total score of the FFMQ-SF was 0.85, and McDonald’s ω was 0.85.

### Sub – health measurement scale version 1.0

The SHMS V1.0 is used to measure suboptimal health levels, which was developed in China based on the concept of subhealth (Xu et al., 2011; Xue et al., 2021). It includes three sub-scales: Physiological Suboptimal Health, Psychological Suboptimal Health and Social Suboptimal Health, comprising a total of nine dimensions and 39 items. PSH consists of 14 items, including factors related to physical condition, organ function, physical activity, and vitality. MSH includes 12 items, encompassing positive emotions, psychological symptoms, and cognitive function. SSH comprises 9 items related to social adaptation, social resources, and social support. Each item includes 5 response options, requiring participants to select the option that best represents their true feelings (rated on a 1–5 scale). Of the 39 items, 19 are reverse-scored. Raw total scores for each dimension, sub-scale, and the overall scale are calculated as the sum of scores from individual items, with higher scores indicating better health. To facilitate understanding and comparison, transformed scores are used for statistical analysis. Transformed scores are calculated as follows: (raw score – theoretical minimum score)/(theoretical maximum score – theoretical minimum score) × 100. In this study, we used the transformed total score of suboptimal health. In this study, the Cronbach’s α for the total score of the SHMS was 0.97, and McDonald’s ω was 0.97.

### The perceived stress scale

This study utilized the Chinese version of the Perceived Stress Scale (CPSS) (Cohen et al., 1983; Li et al., 2021) to measure participants’ perception of psychological stress. The CPSS consists of 14 items, which are divided into two dimensions: Perceived Stress (Items 1, 2, 3, 8, 11, 12, 14) and Perceived Helplessness (Items 4, 5, 6, 7, 9, 10, 13). The scale includes 7 items that are reverse-scored (Items 4, 5, 6, 7, 9, 10, 13). All items are rated on a Likert 5-point scale, ranging from 1 never to 5 very often. Higher scores indicate higher levels of psychological stress. In this study, we used the total score of the CPSS. In this study, the Cronbach’s α for the total score of the CPSS was 0.88, and McDonald’s ω was 0.89.

## Results

### Common method bias test

A common method bias test was conducted using the Harman single-factor test in SPSS 26.0 (Zhou and Long, 2004). The results showed that there were 11 factors with eigenvalues greater than 1, and the first factor accounted for 38.50% of the variance, which was below the critical threshold of 40%. This result indicates that there was no significant common method bias in this study.

### Descriptive statistics and correlation analysis

Table 2 presents the means, standard deviations, and correlations between variables. Social support, mindfulness, perceived stress, and suboptimal health showed significant correlations with each other (|*r*| = 0.38–0.85, *p* < 0.01). Specifically, perceived stress had significant negative correlations with mindfulness, social support, and suboptimal health scores (|*r*| = 0.45–0.85). Mindfulness was positively correlated with social support and suboptimal health scores (*r* = 0.38–0.77), while social support was positively correlated with suboptimal health scores (*r* = 0.65). Table 3 provides detailed information on the correlations between specific dimensions within each scale.

**Table 2:** Results of descriptive statistics and correlation analysis for each variable.

	*M*	*SD*	1	2	3	4	5
1. Gender	1.38	0.49	–				
2. Age	21.85	3.34	−0.06	–			
3. SS	4.15	0.97	−0.05	−0.13	–		
4. FFMQ	66.96	11.08	−0.16^*^	−0.033	0.38^***^	–	
5. CPSS	38.28	10.58	0.17^*^	0.074	−0.45^***^	−0.83^***^	-
6. SHMS	129.34	26.71	−0.11	−0.122	0.65^***^	0.77^***^	−0.85^***^

^*^p < 0.05, ^**^p < 0.01, ^***^p < 0.001.

**Table 3:** Results of correlation analysis for each variable.

	1–1	1–2	1–3	2–1	2–2	2–3	2–4	2–5	3–1	3–2	3–3	3–4	3–5	3–6
1–1 nervousness	1													
1–2 loss of control	0.34^***^	1												
1–3 CPSS-T	0.84^***^	0.80^***^	1											
2–1 Global	−0.62^***^	−0.24^**^	−0.53^***^	1										
2–2 Physical	−0.78^***^	−0.43^***^	−0.75^***^	0.73^***^	1									
2–3 Mental	−0.77^***^	−0.62^***^	−0.85^***^	0.69^***^	0.88^***^	1								
2–4 Social	−0.52^***^	−0.78^***^	−0.78^***^	0.46^***^	0.64^***^	0.81^***^	1							
2–5 SHMS-T	−0.77^***^	−0.62^***^	−0.85^***^	0.70^***^	0.94^***^	0.97^***^	0.84^***^	1						
3–1 Observing	−0.16^*^	−0.57^***^	−0.43^***^	0.12	0.24^**^	0.32^***^	0.55^***^	0.37^***^	1					
3–2 Describing	−0.31^***^	−0.76^***^	−0.64^***^	0.13	0.38^***^	0.49^***^	0.75^***^	0.54^***^	0.65^***^	1				
3–3 Acting with awareness	−0.75^***^	−0.48^***^	−0.76^***^	0.63^***^	0.75^***^	0.78^***^	0.55^***^	0.77^***^	0.18^*^	0.29^***^	1			
3–4 Non-judging	−0.56^***^	−0.18^*^	−0.46^***^	0.49^***^	0.51^***^	0.48^***^	0.26^***^	0.48^***^	0.003	0.09	0.63^***^	1		
3–5 Non-reacting	0.01	−0.52^***^	−0.30^***^	−0.01	0.10	0.17^*^	0.47^***^	0.23^**^	0.58^***^	0.62^***^	0.011	−0.29^***^	1	
3–6 FFMQ_T	−0.61^***^	−0.76^***^	−0.83^***^	0.47^***^	0.66^***^	0.74^***^	0.80^***^	0.77^***^	0.70^***^	0.78^***^	0.73^***^	0.51^***^	0.50^***^	1

^*^p < 0.05, ^**^p < 0.01, ^***^p < 0.001. 1–1 to 1–3 are the subscale and total scale scores of CPSS. Similarly, 2–1 to 2–5 are the parts of SHMS, and 3–1 to 3–6 are the parts of FFMQ.

### Simple mediation analyses

Controlling for gender and age, mindfulness positively predicted suboptimal health scores, *β* = 0.77, *p* < 0.001, with an adjusted *R*^2^ of 0.60, Δ*R*^2^ = 0.58, *F* (1, 169) = 250.17, *p* < 0.001.

**Figure 1:**
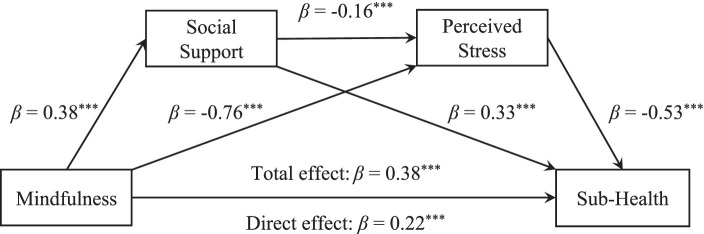
Chain-mediated pathways of social support and stress perception affecting suboptimal health. The prediction coefficient (*β*) and significance are shown in the figure. ^***^ *p* < 0.001.

To assess the chain mediation model, referred to as Model 6, the study employed the PROCESS plugin for SPSS. In this model, mindfulness served as the independent variable, while suboptimal health status was the dependent variable. Social support and perceived stress functioned as the mediators in a sequential chain, with gender and age included as covariates to control for their effects. The relationship dynamics among these variables are illustrated in Figure 1. The results demonstrate that the overall regression model had a significant explanatory power, evidenced by an *R*^2^ value of 0.16 and an *F*(3, 169) = 10.47, with a significance level of *p* < 0.001.

Bootstrap sampling was used to test the indirect effects of mediation. The analysis revealed the following indirect effects: Social support, as a mediator, exhibited an indirect effect of 0.01 (95% CI = [0.20, 0.41]); Perceived stress, as a mediator, demonstrated an indirect effect of 0.04 (95% CI = [0.03, 0.05]); The combined mediation of social support and perceived stress showed an indirect effect of 0.003 (95% CI = [0.001, 0.005]); The total of all indirect effects was 0.05 (95% CI = [0.04, 0.06]). These findings support a chain mediation effect where social support and perceived stress sequentially mediate the positive impact of mindfulness on suboptimal health. Detailed outcomes of the mediation analyses are presented in Table 4.

**Table 4:** The mediating role of social support and stress perception in the influence of mindfulness on sub-health.

Influence path	Indirect effect	Boot SE	95% confidence interval	Relative mediating effect
Total	0.050	0.005	[0.04, 0.06]	–
Mindfulness → SS → SubHealth	0.011	0.002	[0.01, 0.02]	22.00%
Mindfulness → PS → SubHealth	0.036	0.005	[0.03, 0.05]	72.00%
Mindfulness → SS → PS → SubHealth	0.003	0.001	[0.001, 0.005]	6.00%

Further analysis explored the moderating effect to confirm the specificity of the hypothesized chain mediated pathway. Controlling for gender and age, with mindfulness as the independent variable, perceived stress as the mediation variable, social support as the moderating variable, and suboptimal health as the dependent variable (all standardized), Models 8 and 15 were used for moderated mediation analysis.

In Model 8, the interaction of social support and mindfulness on perceived stress was not significant, β = −0.09, 95% CI = [−0.22, 0.05], *p* = 0.21, and the interaction of social support and mindfulness on suboptimal health was not significant, β = −0.03, 95% CI = [−0.13, 0.08], *p* = 0.63. The moderation effect of social support in the first half of the path, where mindfulness influences suboptimal health through perceived stress, was not significant. In Model 15, the interaction of mindfulness and social support on suboptimal health was not significant, β = −0.07, 95% CI = [−0.20, 0.06], *p* = 0.31, and the interaction of social support and perceived stress on suboptimal health was not significant, β = −0.06, 95% CI = [−0.18, 0.05], *p* = 0.30. The moderation index of social support in this model was β = 0.05, 95% CI = [−0.07, 0.13], confirming that social support did not significantly moderate the latter half of the path, where mindfulness influences suboptimal health through perceived stress.

## Discussion

This investigation sought to determine the influence of trait mindfulness on suboptimal health outcomes, as well as to unravel the mechanisms through which trait mindfulness impacts such health conditions. By analyzing the interrelations among psychological constructs like mindfulness, suboptimal health, perceived stress, and social support, we assessed the mediating roles of social support and perceived stress. The findings revealed that higher levels of mindfulness are indeed associated with better suboptimal health outcomes, thus confirming Hypothesis 1. Moreover, the beneficial effect of mindfulness on suboptimal health was found to be mediated through enhanced social support (in line with Hypothesis 2), reduced perceived stress (corroborating Hypothesis 3). Furthermore, the combined mediating effect of both social support and perceived stress were evident (validating Hypothesis 4). Consequently, individuals with elevated trait mindfulness tend to be more attuned to the social support available to them and experience diminished stress, which collectively contributes to a reduction in symptoms of suboptimal health and an improvement in overall health.

The study’s findings underscore the significant role of trait mindfulness in fostering better health outcomes and highlight the pathways through which mindfulness exerts its positive effects. The mediation by social support and perceived stress suggests that mindfulness interventions could be beneficial in enhancing individuals’ social networks and coping mechanisms, thereby reducing the impact of stress on health. These insights pave the way for incorporating mindfulness training into public health strategies aimed at mitigating suboptimal health symptoms and improving well-being. Additionally, the research highlights the importance of psychological resilience factors, such as social support, in the relationship between mindfulness and health, offering avenues for future studies to explore targeted interventions that enhance these protective mechanisms.

### Mindfulness and suboptimal health

As shown in Table 2, there is a moderate to high positive correlation between FFMQ total scores and SHMS total scores. Additionally, mindfulness significantly positively predicts suboptimal health levels. In other words, individuals with higher levels of trait mindfulness tend to observe their internal and external experiences more, label them with words, pay more attention to their current activities, and do not judge or react to their feelings, cognition, and emotions. They may experience fewer physical, psychological, and social suboptimal health symptoms, thus having higher suboptimal health levels. This aligns with previous research findings regarding the alleviating effects of mindfulness on various suboptimal health symptoms (Sharma and Rush, 2014; Bamber and Schneider, 2016; Whitaker et al., 2019). Furthermore, Table 3 shows the correlations between specific facets. Notably, there is a strong correlation between the Observing facet of FFMQ and various dimensions of SHMS. This suggests that being focused on the present moment, rather than acting unconsciously or absentmindedly, is more beneficial for one’s health. However, the Non-reacting facet scores have relatively weaker correlations with various dimensions of SHMS. Individuals with higher scores in this aspect tend to allow sensations, cognitions, and emotions to come and go without being attached or reactive. When revising the Chinese version of FFMQ, the reliability of data related to the Non-reacting facet was lower compared to the American sample. This could be due to differences in how Eastern and Western participants understand mindfulness (Deng et al., 2011). Therefore, overall, it can be concluded that higher levels of trait mindfulness predict higher levels of suboptimal health.

### The mediating role of social support and stress

Firstly, the results of the mediation pathway analysis indicate that perceived social support significantly partially mediates the positive relationship between mindfulness and suboptimal health. Additionally, the serial mediation pathway from social support to stress is also significant. This suggests that participants with higher levels of trait mindfulness are more likely to report higher perceived social support, which is associated with higher suboptimal health levels. Mindfulness enables individuals to focus on their current experiences and be aware of the support they receive from their social networks (Kuhl and Boyraz, 2016). This promotes openness and sharing of emotions with others (Schellekens Melanie et al., 2016). Furthermore, mindfulness emphasizes an open attitude, increasing gratitude and empathy, which can enhance interactions with others and strengthen social relationships (Caputo, 2015), thereby increasing perceived social support (Swickert et al., 2018). Previous research has also explored how mindfulness affects social support (Kuhl and Boyraz, 2016) and the mediating role of social support in mindfulness’s improvement of fatigue (Sun et al., 2019). Moreover, the stress-buffering hypothesis, which this study references, posits that social support further improves health outcomes by regulating stress (Cohen and Wills, 1985). This aligns with the significant serial mediation pathway found in this study. However, given that the effects of mindfulness on both pathways from mindfulness to suboptimal health through social support are relatively small in this study, we offer a cautious interpretation of this aspect of the mechanism. Mindfulness involves the direct awareness of what is happening in the present moment, regardless of whether one’s experiences are positive, negative, or neutral. We suggest that individuals with higher levels of mindfulness tend to perceive and experience all events or stimuli with a more accepting mindset. These individuals do not engage in exaggerating evaluations or overinvolvement with relatively negative stress experiences. Therefore, they experience significantly less stress. In contrast, the perception of positive social support may not significantly differ based on mindfulness levels when compared to perceived stress. This is also reflected in the correlation coefficients between FFMQ and social support and CPSS scores, where the correlation with CPSS is much stronger than that with perceived social support. In summary, the current study’s results provide additional empirical evidence for the mechanisms by which mindfulness influences overall suboptimal health symptoms. This provides insights into the pathways through which mindfulness affects suboptimal health.

Secondly, perceived stress significantly partially mediates the positive relationship between mindfulness and suboptimal health, and the effect size of this pathway is relatively high. The stress-buffering hypothesis of mindfulness has some physiological evidence to support it. Mindfulness has been shown to alter two stress processing pathways in the brain: it increases the recruitment of prefrontal regulatory regions that may inhibit activity in stress processing regions (“top-down” regulatory pathways), and it may directly impact the reactivity of stress processing regions (“bottom-up” reduced stress reactivity pathways) (Creswell and Lindsay, 2014). In other words, mindfulness protects individuals from becoming excessively immersed in stress experiences by reducing stress evaluation and reactions, thus alleviating stress (Sharma and Rush, 2014; Bamber and Schneider, 2016). This ultimately reduces the adverse effects of stress on an individual’s physical and mental health. In addition, social support, through its buffering effect on stress, further improves an individual’s suboptimal health levels.

### Implications

Our study distinguishes itself from previous research in the field by delving more deeply into the effects of mindfulness on sub-health and its subtle mechanisms. Whereas previous studies have broadly linked mindfulness to positive health outcomes, our study goes further by identifying the specific pathways of enhanced perceived social support and reduced stress levels as mediators in this relationship. The present study provides evidence for the directional and sequential chain mediating structure of mindfulness as a predictor of suboptimal health status by testing alternative structural models. Furthermore, the findings emphasize the practical value of mindfulness as a tool for improving health and well-being. Through thoughtful implementation, mindfulness practices can be effectively integrated into a variety of settings, providing an ongoing and efficacious strategy for improving public health. For example, programs such as MBSR can be integrated into healthcare settings and everyday life to provide patients with tools to manage stress and thereby prevent many stress-related health problems.

### Limitations and future research

Although this study contributes to the understanding of the relationships between mindfulness, social support, stress, and suboptimal health by constructing an integrated model and supplementing existing research, it has some limitations. Firstly, while our sample size met the requirements of G-Power standards, it is relatively small. Additionally, we did not differentiate between individuals with and without mindfulness practice experience. Future research can further expand on this by incorporating participant perspectives. Secondly, in terms of measurement tools, we used only one self-developed item to measure perceived social support, which may have limitations. Future studies can further validate the mediation pathways of social support using commonly used social support assessment scales. Thirdly, it should be noted that there was no mindfulness intervention in this study. Future research could conduct a different duration of mindfulness intervention study to further validate the results of this study. Finally, most of the chain mediation in this study involves partial mediation, indicating that there may be other underlying mechanisms in the relationship between mindfulness and suboptimal health. Future research needs to continue exploring these mechanisms.

In conclusion, the present study showed that the cultivation of trait mindfulness may be advantageous for individuals’ sub-health, and perceived social support and perceived stress are important mechanisms underlying this effect.

## Data availability statement

The raw data supporting the conclusions of this article will be made available by the authors, without undue reservation.

## Ethics statement

The studies involving humans were approved by the Institutional Review Board (or Ethics Committee) of The Institute of Psychology, Chinese Academy of Sciences. The studies were conducted in accordance with the local legislation and institutional requirements. The participants provided their written informed consent to participate in this study.

## Author contributions

JL: Conceptualization, Investigation, Data curation, Writing – original draft. XZ: Conceptualization, Formal analysis, Methodology, Validation, Visualization, Writing – original draft, Writing – review & editing. YW: Conceptualization, Investigation, Supervision, Validation, Writing – review & editing.

## Footnotes

^1^https://www.wjx.cn/
